# *Pcdh11x* controls target specification of mossy fiber sprouting

**DOI:** 10.3389/fnins.2022.888362

**Published:** 2022-09-01

**Authors:** Wenshu Luo, Natalia Andrea Cruz-Ochoa, Charlotte Seng, Matteo Egger, David Lukacsovich, Tamás Lukacsovich, Csaba Földy

**Affiliations:** Laboratory of Neural Connectivity, Brain Research Institute, Faculties of Medicine and Science, University of Zürich, Zurich, Switzerland

**Keywords:** granule cell, axonal rewiring, mossy fiber sprouting, cell adhesion molecule, synaptic adhesion molecule, protocadherin, *Pcdh11x*, target specificity

## Abstract

Circuit formation is a defining characteristic of the developing brain. However, multiple lines of evidence suggest that circuit formation can also take place in adults, the mechanisms of which remain poorly understood. Here, we investigated the epilepsy-associated mossy fiber (MF) sprouting in the adult hippocampus and asked which cell surface molecules define its target specificity. Using single-cell RNAseq data, we found lack and expression of *Pcdh11x* in non-sprouting and sprouting neurons respectively. Subsequently, we used CRISPR/Cas9 genome editing to disrupt the *Pcdh11x* gene and characterized its consequences on sprouting. Although MF sprouting still developed, its target specificity was altered. New synapses were frequently formed on granule cell somata in addition to dendrites. Our findings shed light onto a key molecular determinant of target specificity in MF sprouting and contribute to understanding the molecular mechanism of adult brain rewiring.

## Introduction

Mossy fiber (MF) sprouting in the hippocampal dentate gyrus represents a non-developmental form of circuit formation in the adult brain (for review, see Seng et al., 2022). MF sprouting has been extensively studied in the context of temporal lobe epilepsies (Noebels et al., 2012) and is inducible by mechanical (Laurberg and Zimmer, 1981; Zimmer and Gähwiler, 1987), electrical (Sutula et al., 1988), chemical (Tauck and Nadler, 1985), and genetic approaches (Luo et al., 2021). During MF sprouting, granule cells (GCs) grow new axonal branches into the inner molecular layer (IML) of dentate gyrus and form synapses mostly on proximal dendrites of GCs (Laurberg and Zimmer, 1981; Wenzel et al., 2000; Cavazos et al., 2003; Frotscher et al., 2006; Luo et al., 2021), but potentially also on interneurons as observed in chronically epileptic rats (Frotscher et al., 2006). This new circuit is formed on top of the developmentally established MF circuit, which extends into CA3 (Hainmueller and Bartos, 2020). As any neuronal wiring, MF sprouting is thought to require molecular programs for axon growth, target specification, and synapse formation (Godale and Danzer, 2018; Koyama and Ikegaya, 2018; Luo et al., 2021). Such processes generally involve synaptic cell-surface receptors and cell-adhesion molecules (Missaire and Hindges, 2015; de Wit and Ghosh, 2016; Sanes and Zipursky, 2020; Südhof, 2021), which hereafter we collectively refer to as CAMs, for short.

The role of different CAMs during developmental MF wiring is relatively well understood. Netrin and slit signaling control axon guidance toward CA3 (Muramatsu et al., 2010). Plexin and semaphorin signaling establish layer specificity within CA3 (Chen et al., 2000; Suto et al., 2007; Tawarayama et al., 2010). Other CAMs regulate MF target specificity and/or synapse function to CA3 pyramidal cells (NCAM, Cdh9, Gpr158) (Cremer et al., 1998; Williams et al., 2011; Basu et al., 2017; Condomitti et al., 2018), interneurons (Kirrel3, Igsf8) (Martin et al., 2015; Apóstolo et al., 2020), or possibly to both (Pcdh19) (Hoshina et al., 2021). Finally, semaphorin-neuropilin-plexin (Bagri et al., 2003) and ephrin (Xu and Henkemeyer, 2009; Liu et al., 2018) signaling control MF pruning. By contrast, CAM signaling in MF sprouting is much less understood. While abundance changes in multiple CAMs have been reported in models of temporal lobe epilepsy or directly in sprouting fibers, their involvement in sprouting remains elusive (see section “Discussion”). Recently, we studied transcriptomic mechanisms of MF sprouting and identified a transcriptomic regulator, Id2, whose sole overexpression in GCs induced MF sprouting (Luo et al., 2021). While Id2-induced MF sprouting alone was insufficient to provoke pathological network activity seen in epilepsy, further lessening its potential as a clinical target (Buckmaster, 2014), MF sprouting remains a robust model for studying circuit formation in the adult brain.

Here, we used single-cell RNA-seq data generated using the intrahippocampal kainic acid- (KA) injection model to study CAMs in MF sprouting. We used the KA, but not Id2, model because MF sprouting develops significantly faster by KA (within weeks) than by Id2 (requires months) (Luo et al., 2021). Thus, functional testing, which here we aimed for, is more attainable in the KA model. We focused on differentially expressed CAMs, and identified three candidate genes–*Fat3*, *Cntn4* and *Pcdh11x*–which were upregulated after sprouting. We targeted these genes by CRISPR/Cas9 guide RNAs (gRNAs) to disrupt their genomic sequences in GCs *in vivo*, and confirmed mutation/deletions in *Pcdh11x*, likely rendering this gene null mutant in most cells. After GC-specific *Pcdh11x* KO, KA still induced MF sprouting, but new synapses frequently and atypically formed on GC somata.

## Materials and methods

### Animals

All animal protocols and husbandry practices were approved by the Veterinary Office of Zurich Kanton. The University of Zurich animal facilities comply with all appropriate standards (cages, space per animal, temperature, light, humidity, food, and water) and cages were enriched with materials that allow the animals to exert their natural behavior. The following lines were used in this study: Calb1-Cre: B6;129S-Calb1^TM 2^.^1(cre)Hze^/J, JAX:028532 and H11-LSL-Cas9: B6;129-Igs2tm1(CAG-cas9*)Mmw/J, JAX:026816. The animals used in this study were obtained by mating the homozygous Calb1-Cre mice with heterozygous H11-LSL-Cas9 mice. In each experiment, the control and non-control animals were littermates.

### List of CAMs

An extended set of 421 CAMs was used for gene expression analysis. We used a previously published list of 406 CAMs (Földy et al., 2016), to which *Nptxr*, *Sema3a*, *Sema3c*, *Sema3d*, *Sema3g*, *Sema4a*, *Sema4b*, *Sema4c*, *Sema4f*, *Sema5a*, *Sema5b*, *Sema6a*, *Sema6b*, *Sema6c*, *Slit1*, *Slit2*, *Slit3* were added, whereas *Ptpn2* and *Ptpn5* were removed as non-receptor type protein tyrosine phosphatases.

### Design of guide RNAs

To design guide RNAs (gRNAs), we prioritized to (i) target early coding regions that are shared by all transcript variants of a gene in order to maximize the probability of introducing functionally disabling mutations and/or deletions, (ii) minimize the possibility of unwanted off-target effects, and (iii) maximize editing efficacy at the intended target site. For each targeted gene (i.e., *Fat3*, *Cntn4*, and *Pcdh11x*), two gRNAs (19 - 21 bp long) were designed targeting possible target sites on exons 1-3, followed by a 3 bp long NGG PAM sequence on the 3′ end (see Supplementary Figure 1A for specific sequences). Each gRNAs were evaluated by “CRISPR-Cas9 gRNA checker” (Integrated DNA technologies, Inc.), resulting in two scores: (1) “on-target score” that indicates the predicted editing performance of gRNA at the intended target site (higher value indicates better performance) and (2) “off-target score” that indicates potential off-target effects and N (number) nucleotide mismatch hits during genome screening (in a range from 0 to 100, higher value indicates lower off-target risk). *Fat3-gRNA1*: on-target score 66, off-target score 36 (high off-target risk), 0 mismatch only on *Fat3*, no potential off-target sites were identified with 1 or 2 mismatches. *Fat3-gRNA2*: on-target score 36 (low on-target performance), off-target score 82, 0 mismatch hit only on *Fat3*, no potential off-target sites with 1 mismatch, one 2 mismatch off-target site were found in a non-coding region (chr5: + 18040716). *Cntn4-gRNA1*: on-target score 40 (low on-target performance), off-target score 86, 0 mismatch hit only on *Cntn4*, no potential off-target sites with 1 mismatch, one 2 mismatch off-target site were found in a non-coding region (chr6: + 8683610). *Cntn4-gRNA2*: on-target score 8 (low on-target performance), off-target score 58, 0 mismatch hit only on *Cntn4*, no potential off-target sites with 1 or 2 mismatches. *Pcdh11x-gRNA1*: on-target score 56, off-target score 85, 0 mismatch only on *Pcdh11x*, no potential off-target sites with 1 or 2 mismatches. *Pcdh11x-gRNA2*: on-target score 53, off-target score 72, 0 mismatch hit only on *Pcdh11x*, no potential off-target sites with 1 or 2 mismatches. Note that the gRNA’s on-target performance was subsequently tested and validated in cell cultures before *in vivo* experiments (see below).

### Plasmids and viruses

For *in vivo* genomic targeting of CAMs, gRNAs were designed and cloned into pBSK-U6 backbone (pBSK-U6-gRNAs). The plasmids were purified and used for evaluation of knockout efficiency in cell culture. After evaluation, the same gRNAs were cloned into Cre-dependent tRFP expression vector and packaged into adeno-associated virus (AAV) serotype DJ/8. For *Fat3* targeting, a viral mixture (2.2 × 10^13^ vg/ml) of vWL51.AAVDJ8/2-[hU6-gRNA1(mFat3)]rev-hSyn1- dlox-TurboRFP(rev)-dlox-WPRE-hGHp(A) and vWL52.AAV DJ8/2-[hU6-gRNA2(mFat3)]rev-hSyn1-dlox-TurboRFP(rev)- dlox-WPRE-hGHp(A) were used. For *Cntn4* targeting, a viral mixture (1.7 × 10^13^ vg/ml) of vWL44.AAVDJ8/2-[hU6-gRNA1(mCNTN4)]rev-hSyn1-TurboRFP(rev)-WPRE-hGHp(A) and vWL45.AAVDJ8/2-[hU6-gRNA2(mCntn4)]rev-hSyn1-TurboRFP(rev)-WPRE-hGHp(A) were used. For *Pchd11x* targeting, a viral mixture (1.7 × 10^13^ vg/ml) of wWL46.AAVDJ8/2-[hU6-gRNA1(mPcdh11x)]rev-hSyn1- TurboRFP(rev)-WPRE-hGHp(A) and vWL47.AAVDJ8/2-[hU6-gRNA2(mPcdh11x)]rev-hSyn1-TurboRFP(rev)-WPRE-hGHp(A) were used. All viral vectors were produced by the Viral Vector Facility (VVF) of the Neuroscience Center Zurich (ZNZ).

### Validation of CAM targeting guide RNAs in cell culture

The mixture of gRNA expressing vectors (0.4 μg of pBSK-U6-gRNA1 and 0.4 μg of pBSK-U6-gRNA2) were transfected into Neuro-2a cells expressing doxycycline-inducible CRISPR Cas9 nuclease from Rosa26 locus (GeneCopoeia, SL508) using Lipofectamine 3000, according to recommendations of the manufacturer (Invitrogen). Forty-eight hours after transfection, doxycycline (1 μg/ml) was applied to induce stable Cas9 expression. To maintain Cas9 expression, the medium containing doxycycline was renewed every 48 h. Cells were harvested 7 days after transfection and prepared for Sanger sequencing.

### Stereotaxic injection

Mice were deeply anesthetized and placed into a stereotactic apparatus. Microinjections were performed at a rate of 100 nl/min using a programmable syringe pump with a 35-gauge beveled NanoFil needle (World Precision Instruments, United States). For *in vivo* CAM targeting, 500 nl of the above mentioned viruses were injected into the ventral dentate gyrus (−3.4 mm anterior/posterior, 2.9 mm middle/lateral, −3.3 mm ventral/dorsal to bregma). To induce MF sprouting, 70 nl of KA (5 mM) was injected into the same position 4 weeks later or into gRNA non-injected animals.

### *In vitro* electrophysiology

Brain slice preparation, recording solutions, whole-cell patch-clamp recording, and measurement of biophysical properties were as previously described (Luo et al., 2021). In short, neurons were visualized by infrared differential interference contrast optics in an upright microscope (Olympus; BX-51WI) using Hamamatsu Orca-Flash 4.0 CMOS camera and recorded using borosilicate glass pipettes with filament (Harvard Apparatus; GC150F-10; o.d. 1.5 mm; i.d. 0.86 mm; 10-cm length). Recordings were made using MultiClamp700B amplifier (Molecular Devices), signals were filtered at 10 kHz (Bessel filter) and digitized (50 kHz) with a Digidata1440A and pClamp10 (Molecular Devices). Spontaneous events were recorded in voltage clamp mode at -60 mV for 5 min, in presence of Gabazine (10 μM), or APV (10 μM) and NBQX (5 μM). The data analysis was performed using Python, R, Clampfit (Molecular Devices), and MiniAnalysis. For subsequent *post hoc* visualization, cells were filled with biocytin (Sigma-Aldrich, 2%) during recording. For all electrophysiological experiments, the experimenter was blind to the recording condition.

### Histology

#### Sample preparation

Animals were deeply anesthetized and transcardially perfused first with 3 ml 0.9% saline solution followed by 3 ml 0.1% Na_2_S in 0.1 M PB solution, and then by 4% paraformaldehyde (PFA) in 0.1 M PB (1ml/1g bodyweight). Brains were immersed into 4% PFA in 0.1 M PB overnight at 4°C and then sectioned the next day using a vibratome, or further transferred into 30% sucrose in 0.1 M PB and stored at 4°C until sectioning using a frozen tissue sliding microtome. Fixed brains were cut into 50 or 80 μm thick horizontal sections.

#### Immunohistochemistry

Slices were first permeabilized and blocked in incubating medium (0.1 M PB containing 5% normal goat serum and 0.2% Triton) for 1 hour at room temperature, and then incubated overnight with primary antibodies at 4°C. Primary antibodies used: rabbit monoclonal anti-SLC30A3 (ZnT3; ThermoFisher, PA5-77769, 1:600), guinea pig polyclonal ZnT3 antiserum (Synaptic system, #197004, 1:500), rabbit polyclonal PCDH11X antibody (aa987-1117, LS-C673568, LifeSpan BioSciences, 1:500). Next day, slices were rinsed in 0.1 M PB and incubated with secondary antibodies overnight at 4°C. Secondary antibodies used: goat anti-rabbit IgG (H + L) cross-adsorbed, Alexa Fluor 488 (Invitrogen, A-11008, 1:500), anti-guinea pig IgG (H + L) highly cross-adsorbed secondary antibody, Alexa Fluor 568 (Invitrogen, A-11075, 1:500). Sections were rinsed in 0.1 M PB (some sections were subsequently stained with DAPI for nuclear staining) and mounted in Vectashield (Vector Laboratories) for analysis.

#### Timm’s staining

Sections were rinsed in 0.1 M PB and post-fixed in 2.5% glutaraldehyde in 0.1 M PB solution for 10 min. Then, sections were rinsed in 0.1 M PB and immersed in Timm’s reaction solutions, a 12:6:2 mixture of 20% gum arabic, hydroquinone, and citric acid trisodium citrate buffer, with 100 μl of 17% silver nitrate solution. The reaction was carried out for 20–30 min at 29°C, then slices were washed thoroughly in 0.1 M PB. After dehydration steps, the sections were mounted using DPX mounting medium and imaged using a Leica wide-field microscope.

#### Morphological reconstruction

Biocytin-filled cell-containing brain slices were fixed 4% PFA in 0.1 M PB overnight at 4°C. Next day, DAB staining (Vectastain ABC KIT, Vector Laboratories) was performed, and sections were dehydrated and mounted in DPX mounting medium (Electron Microscopy Science, United Kingdom). Cells were reconstructed using Neurolucida (MicroBrightField, Inc., United States).

### Image analysis and quantification

Fluorescent images were acquired using Leica Stellaris 5 confocal microscope. Image analyses and quantification were performed in Fiji (version 2.0.0-rc-68/1.52h).

#### Quantification of PCDH11X immunostaining in wild-type animals after kainic acid injection

Tile-scan confocal images (1,024 × 1,024 pixels, zoom 0.75) were obtained using 20x immersion lens (0.75 NA). The mean gray value of PCDH11X immunostaining signals were measured in hilus, granule cell layer (GCL), inner molecular layer (IML), and middle/outer molecular layer (MML/OML). In addition, the mean gray value of PCDH11X immunostaining signal was measured in an area (that is below CA3 and outside hilus) that appeared to be PCDH11X negative in all conditions, to be used as baseline. Then, GCL, IML, and MML/OML signal intensities were normalized by subtracting this baseline signal intensity. In this manner, two images per animal were analyzed, the average values of which are shown in figure(s). As controls, normalized mean gray values from sections collected from ipsi- and contralateral hippocampus of saline injected animals (6 or 10 days after saline) and from the contralateral hippocampus of KA injected animals (6 or 10 days after KA) were averaged and used.

#### Quantification of PCDH11X immunostaining after *Pcdh11x*^Control+KA^ and *Pcdh11x*^KO+KA^

To confirm the location of injections and sufficient delivery of gRNAs into GCs, we included a turboRFP (tRFP) sequence in gRNA expression vectors. As intended, the tRFP signal broadly labeled GCs. However, we also found that the tRFP signal was strong and cross-bleed into the GFP channel to be used for detection of PCDH11X signals. This effect was most prominent in GCL where GC somata were strongly labeled with tRFP. To alleviate this problem, we exposed sections to light for several hours to bleach the tRFP signal and stained them for PCDH11X only afterward. While this treatment lowered the tRFP intensity, it did not completely eliminate the tRFP signal from the GFP channel. We then quantified PCDH11X signal intensity with and without normalization for the tRFP signal seen in the GFP channel. For quantification, tile-scan confocal images (1,024 × 1,024 pixels, zoom 0.75) were obtained using 20x immersion lens (0.75 NA). To obtain tRFP-non-normalized values, we used the same approach as described above (see *Quantification of PCDH11X immunostaining in wild-type animals after KA injection*). To obtain tRFP-normalized values, we first measured signal intensity in the GFP channel in hilus, GCL, IML, MML, and OML separately in sections from *Pcdh11x*^Control^ and *Pcdh11x*^KO^ animals. We chose to do this in KA-non-injected samples, because in these the tRFP signal in the GFP channel was similar to those in KA-injected samples, but the PCDH11X signal was expected to be the lowest. Then, these values were averaged between *Pcdh11x*^Control^ and *Pcdh11x*^KO^ in each region separately (i.e. hilus, GCL, IML, MML/OML), to be used as baselines. Subsequently, these baseline values were subtracted from PCDH11X signal intensities measured in each area (i.e., hilus, GCL, IML, MML/OML) from KA-injected *Pcdh11x*^Control+KA^ and *Pcdh11x*^KO+KA^ animals. In this manner, two images per animal were analyzed, the average values of which are shown in figure(s). Independently of the approach used (i.e., tRFP-normalization or tRFP-non-normalization), PCDH11X signal intensities were significantly lower in GCL and IML of *Pcdh11x*^KO+KA^ samples compared to *Pcdh11x*^Control+KA^ samples.

#### Quantification of Timm’s staining intensity

Bright-field images were acquired using a THUNDER (Leica) wide-field microscope using 40x lens (0.95 NA). Using Fiji, the mean gray value of Timm signals were measured in both GCL and IML, from which the GCL/IML ratio of gray values was calculated. The average value from 4 sections per animal was shown in the plot.

#### Quantification of ZnT3-positive puncta surrounding GC somata

Single panel confocal images (1,024 × 1,024 pixels) were obtained using 63× oil lens (1.4 NA). ZnT3 + signals on 95-170 GC somata from at least 2 images were quantified per animal. The percentage of GC somata surrounded by different numbers of ZnT3 + puncta was calculated based on the surrounding ZnT3 + puncta numbers per soma and total number of somata analyzed.

### Immuno-electron microscopy

After fixation, brains were cut into 80 μm thick sections using a vibratome. For better penetration of the antibodies, single sections were frozen/thawed in liquid nitrogen using sucrose as cryoprotectant with the following concentration steps 10, 20, 30, 20, 10% and washed several times in 0.1 M PB. Then, the sections were treated with 0.5% NaBH_4_ to bind free aldehyde groups for 15 min, followed by 5 min treatment with 3% H_2_O_2_ and 10% Methanol in 0.1 M PB to reduce endogenous peroxidase. After thoroughly washing in 0.1 M PB, the sections were blocked for 1 h at room temperature in 5% normal goat serum in 0.1 M PB and then incubated in rabbit monoclonal anti-SLC30A3 (ZnT3; ThermoFisher, PA5-77769, 1:600) at 4°C overnight. Next day, sections were incubated in biotinylated anti-rabbit solution (1:100, Vector Laboratories) at 4°C overnight. Next day, sections were developed with a standard avidin-biotin peroxidase kit (1:500; Vectastain) and postfixed in 1% OsO_4_ followed by 3 × 5 min washing in 0.1 M PB. After washing, sections were dehydrated and embedded in durcupan (Sigma-Aldrich) and re-sectioned. Finally, 60 nm ultra-thin sections were contrasted with 3% Lead citrate (Leica) and imaged using a FEI Tecnai G2 Spirit transmission electron microscope or Apreo VS (Thermo Fisher Scientific) scanning electron microscope. 3D rendering was performed with Fiji/ImageJ.

### Statistical analyses

Statistical analyses were performed using Prism 9. All values represent mean ± standard error of the mean (SEM). The significance of differences was assessed using Welch’s *t*-test, Mann–Whitney *U* test, one-way ANOVA, or two-way ANOVA, whichever is applicable (noted in text and/or figure legends). Data distribution normality was tested by Shapiro–Wilk Test. For normal distributions, Welch’s *t*-test was performed. For non-normal distributions, non-parametric Mann–Whitney *U* test were performed. Significant main effects or interactions were followed up with *post hoc* testing using the original FDR method of Benjamini and Hochberg. The threshold for significance was *p* = 0.05 or FDR = 0.05, with a precise *p* value stated in each case. Non-significance is indicated with ‘ns’. All tests were two-sided. Data analyses and quantifications were done blindly with respect to treatment.

## Results

### CAM expression changes during mossy fiber sprouting

**FIGURE 1:**
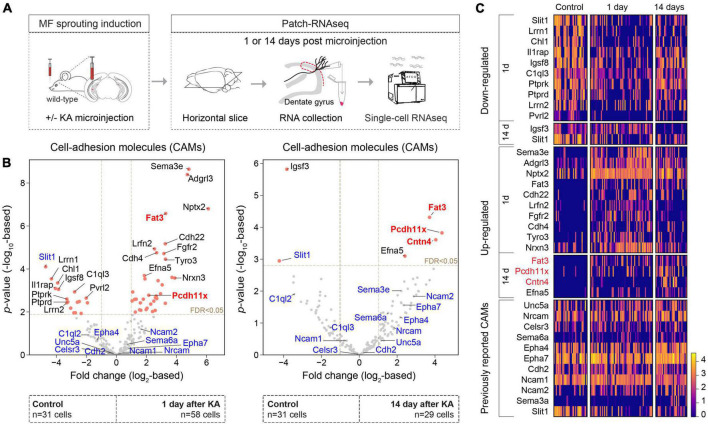
CAM expression changes during MF sprouting. **(A)** Experimental design and schedules used for generating the single-cell RNAseq dataset (Luo et al., 2021; GSE 161619). **(B)** Volcano plots show differentially expressed CAMs in GC 1 **(left panel)** and 14 days **(right panel)** after intrahippocampal KA injection compared to controls. Red points denote differentially expressed genes (FDR < 0.05 and fold change > 2). Gene names highlighted with blue were previously reported in epilepsy and/or MF sprouting models (see section “Discussion”). Gene names highlighted with red were shortlisted for further analysis in this study. **(C)** Heat map of top 10 differentially expressed genes and genes/molecules previously reported in epilepsy and/or MF sprouting models. Scale bar shows log2-normalized gene expression level.

To begin, we further analyzed our previously published single-cell transcriptomic data set consisting of control GCs as well as GCs 1 and 14 days after unilateral hippocampal KA injection (Figure 1A) (Luo et al., 2021; GSE 161619). Based on an extended list of 421 CAMs (see Földy et al., 2016 and section “Materials and methods”), we considered differentially expressed genes (fold change > 2 and FDR < 0.05) between the control and KA data sets (Figure 1B). This analysis revealed significant enrichment of *Fat3, Pcdh11x* in KA GCs, both 1 and 14 days after KA injection. *Fat3*, an atypical cadherin, has been implicated in the development of neuronal morphology (Deans et al., 2011; Krol et al., 2016). *Pcdh11x*, a delta1-type protocadherin, has been implicated in homophilic *trans* cell-cell interactions (Harrison et al., 2020; Pancho et al., 2020), dendritic branching (Wu et al., 2015), and neuronal stem cell differentiation and proliferation (Zhang et al., 2014). We shortlisted these molecules for further analysis. Although *Cntn4* was significantly enriched only in 14-day KA GCs after MF sprouting has developed, we also shortlisted this gene, because it has been linked to circuit formation (Oguro-Ando et al., 2017), target specification (Osterhout et al., 2015), synaptic plasticity (Oguro-Ando et al., 2021), neurodevelopmental disorders (Baig et al., 2017; Oguro-Ando et al., 2017), and Alzheimer’s disease (Carrasquillo et al., 2009). In addition, we looked for CAMs, whose abundance change has been reported in different temporal lobe epilepsy models or in MF sprouting (see section “Discussion,” Figure 1C). However, with the exception of *Slit1* (previously reported to be up-regulated in hippocampal tissue, but down-regulated in KA GCs), their expression did not significantly change in our single-cell data (Figures 1B,C).

### Genomic targeting of CAMs in adult granule cells

**FIGURE 2:**
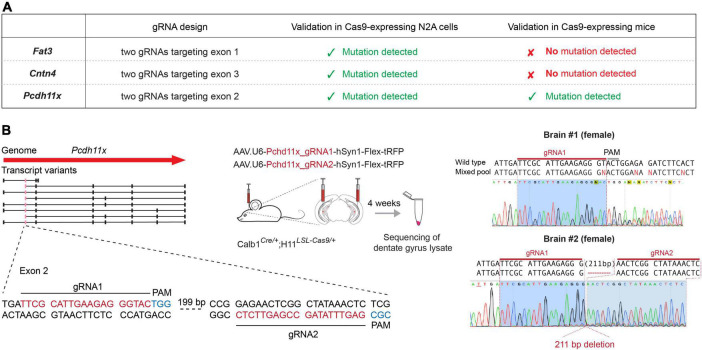
Genomic targeting of CAMs in adult GCs. **(A)** Schematic representation of the genome targeting approach for *Fat3*, *Cntn4*, and *Pcdh11x*. Table shows targeted exons and the presence or absence of mutations during cell culture (Cas9-expressing N2A cells) and *in vivo* (Cas9-expressing mice) validation. **(B)** *In vivo* genomic targeting of *Pcdh11x*. Left panel shows specific gRNA design and experimental schedule. Right panel shows sequence maps of detected mutations and/or deletions in dentate gyrus lysates prepared from two different mice.

To study the role of *Fat3*, *Pcdh11x*, and *Cntn4* in MF sprouting, we aimed to introduce loss-of-function deletions and/or mutations in their genomic sequences. To achieve this goal, we designed two CRISPR/Cas9 guide RNAs (gRNAs) targeting each gene, to be delivered into GCs in the adult brain (Figure 2A and Supplementary Figure 1A, and section “Materials and methods”). To identify the transfected area and neurons that expressed the gRNA, we also included a Cre-dependent turboRFP (tRFP) reporter into the gRNA-expressing AAV vectors. This *in vivo* gene editing approach minimized unwanted effects during development, ensured cell type-specificity and—since *Fat3*, *Pcdh11x*, and *Cntn4* transcripts were virtually absent from control GCs (Figure 1C)—that loss-of-function effects would manifest themselves only after KA injections.

First, we tested gRNAs targeting each gene in cell cultures and confirmed their efficacy in introducing genomic mutations (Supplementary Figure 1). Second, to achieve GC-specific gene manipulations, we separately delivered the pairs of gRNAs into the dentate gyrus of 2 months old *Calb1*^Cre/+^*;*H11*^*LSL*–*Cas*9/+^* mice, in which GCs expressed Cas9. Four weeks later, we confirmed broad presence of the tRFP reporter in the dentate gyrus and prepared lysates for target gene specific PCR amplification. Genomic sequence analysis revealed multiple mutations or large deletions (>200 basepair) in *Pcdh11x*, likely rendering this gene null mutant (KO) in most neurons (Figure 2B and Supplementary Figure 1). By contrast, *Fat3* and *Cntn4* sequences did not display deleterious effects. To further test these two genes, we sequenced 24 single clones from the PCR product of each. This analysis revealed insertions/deletions only in 1/24 of Fat3 and 2/24 of Cntn4 clones, further confirming their inefficient targeting *in vivo* (Supplementary Figure 1). Variations in the *in vivo* targeting efficiency of different genes were not completely unexpected, however, based on these results we could proceed further only with *Pcdh11x*.

### PCDH11X protein levels in the dentate gyrus

**FIGURE 3:**
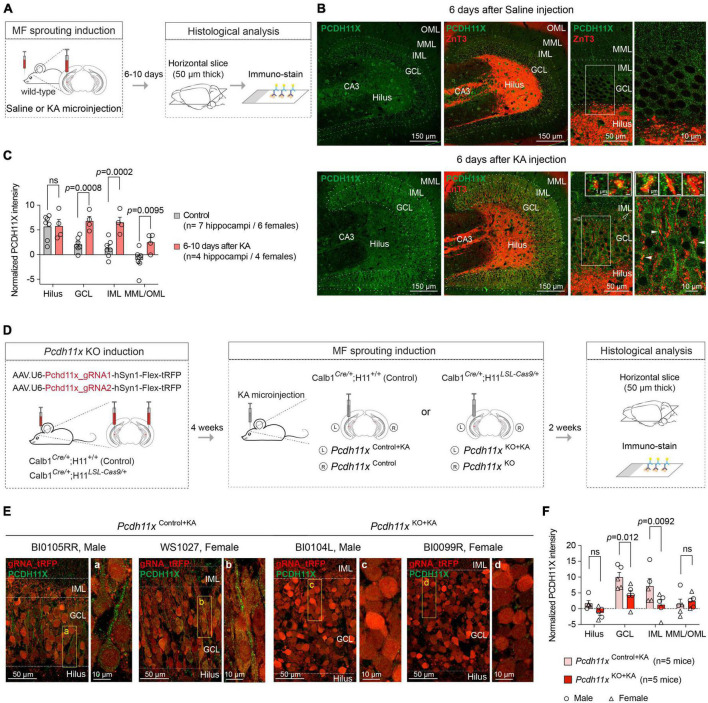
PCDH11X protein expression in the dentate gyrus. **(A)** Schematic representation of the experimental design. **(B)** Confocal images show PCDH11X and ZnT3 immunostaining in the dentate gyrus 6 days after saline (upper row) and KA injection (lower row). From left to right, PCDH11X immunostaining, PCDH11X and ZnT3 immunostaining, higher magnification images of the PCDH11X and ZnT3 immunostaining, and finally higher magnification images of the regions highlighted with white frames in the previous panels are shown. In the lower right panels, inserts show PCDH11X localization in ZnT3 + MF boutons in IML (empty arrowheads) and in GCL (white arrowheads). **(C)** Quantification of the PCDH11X signal in controls and 6–10 days after KA (two-way ANOVA, F_*Layer*_ (3,27) = 17, *p* < 0.0001; F_*Treatment*_ (1,9) = 14, *p* = 0.0051; F_*Layer x Treatment*_ (3,27) = 5.1, *p* = 0.0066; *post hoc* analyses: control vs KA, hilus: ns, *p* = 0.94; GCL: *p* = 0.0008; IML: *p* = 0.0002; MML/OML: *p* = 0.0095). **(D)** Schematic representation of the experimental design in Cas9-expressing and -non-expressing mice. **(E)** Confocal images show PCDH11X immunostaining in the dentate gyrus of *Pcdh11x*^Control+KA^ and *Pcdh11x*^KO+KA^ mice 14 days after KA. Areas highlighted with boxes are shown in higher magnification in panels **(a–d)**. **(F)** Quantification of tRFP-normalized PCDH11X levels in dentate gyrus of *Pcdh11x*^Control+KA^ and *Pcdh11x*^KO+KA^ mice [two-way ANOVA, F_*Layer*_ (3,24) = 19, *p* < 0.0001; F_*KO*_ (1,8) = 3.7, *p* = 0.091; F_*Layer x KO*_ (3,24) = 5.5, *p* = 0.0049; *post hoc* analyses: *Pcdh11x*^Control+KA^ vs *Pcdh11x*^KO+KA^, hilus: ns, *p* = 0.17; GCL: *p* = 0.012; IML: *p* = 0.0092; MML/OML: *p* = 0.62] (for tRFP-non-normalized values see Supplementary Figure 2).

To investigate the role of *Pcdh11x* in MF sprouting, we first aimed to establish the extent of PCDH11X protein expression (we refer to protein form with capitalized gene name) in the dentate gyrus of wild-type animals, including if cell types other than GCs expressed this protein. Using PCDH11X antibody, we immunostained sections 6–10 days after saline- and KA-injections (Figures 3A,B and Supplementary Figure 2). We presumed that *Pcdh11x* mRNA seen 1 day after KA (Figure 1) would be translated and detectable by this time. In addition, 6–10 days after KA likely represents a critical period for establishing MF target specificity, since most growing axons would still advance toward IML during this phase (MF sprouting starts ∼2–3 days after KA and becomes largely established ∼14 days after KA) (Luo et al., 2021).

In controls, some cells in the hilus, GCL and dentate molecular layers appeared to be PCDH11X positive and a weak punctate, possibly background signal, could be observed in all dentate layers (Figure 3B and Supplementary Figure 2). About 6–10 after KA, less hilar but more GCL cells were PCDH11X positive, and a prominent punctate PCDH11X signal became apparent in GCL and IML (Figures 3B,C). In part, the emergence of this signal was due to PCDH11X located in the somato-dendritic domain of GCs (Figure 3B). In addition, PCDH11X appeared to localize in zinc transporter-3 (ZnT3, a frequently used MF marker) positive MF boutons in IML and GCL (Figure 3B, inserts in lower right panels). These results thus revealed KA-induced PCDH11X enrichment in areas relevant for MF sprouting and during a phase likely critical for target specification. However, the question whether PCDH11X enrichment originated only from GCs or possibly also from other cells expressing this protein remained open. To answer this question, we used the above described Cas9 system to evaluate if genetic *Pcdh11x* KO in GCs occluded KA-induced PCDH11X enrichment.

We injected *Pcdh11x* targeting gRNA- and tRFP-containing AAVs into the ventral dentate gyrus of *Calb1*^Cre/+^ (*Pcdh11x*^Control^, lacking Cas9 expression) or *Calb1^Cre/+^;*H11*^*LSL*–*Cas*9/+^* mice (*Pcdh11x*^KO^). Four weeks later, we injected KA into the left dentate gyrus to induce *Pcdh11x* upregulation and MF sprouting, and then two weeks later, we prepared 50 μm thick horizontal sections for histological analysis (Figure 3D). Using the tRFP reporter, we localized the transfected area and quantified the ratio of tRFP + and DAPI + cells in GCL, which revealed > 90% transfection efficacy both conditions (*Pcdh11x*^Control+KA^: 92.4 ± 0.97%, n = 3; *Pcdh11x*^KO+KA^: 92.82 ± 0.49%, n = 3) showing that our manipulations broadly impacted GCs. Using immunostaining, we then examined PCDH11X protein expression 14 days after KA injection. We found that the punctate and in some cells somato-dendritic PCDH11X labeling was present in *Pcdh11x*^Control+KA^ (following the same pattern as in wild-type animals 6 days after KA), but largely absent from *Pcdh11x*^*KO* + *KA*^ samples (Figure 3B). However, in both *Pcdh11x*^Control+KA^ and *Pcdh11x*^*KO* + *KA*^ samples, we also noticed that the tRFP signal used for cell labeling (intended to be visible only in RFP channel) was intense and visible in the GFP channel used for PCDH11X detection. This effect was most prominent in GCL where GC somata are located. To address this issue, we quantified PCDH11X signals with and without normalization to tRFP seen in the GFP channel (see section “Materials and methods”). Independently of the normalization approach used, PCDH11X signal intensity was significantly lower in GCL and IML in *Pcdh11x*^KO+KA^ compared to *Pcdh11x*^Control+KA^ samples (tRFP-normalized, *Pcdh11x*^Control+KA^: hilus: 1.7 ± 0.88, GCL: 9.97 ± 1.57, IML: 7.17 ± 2.39, MML/OML: 1.33 ± 1.68, n = 5; *Pcdh11x*^KO+KA^: hilus: -1.28 ± 0.81, GCL: 4.30 ± 1.49, IML: 1.27 ± 1.57, MML/OML: 2.40 ± 0.98, n = 5, Figures 3E,F) (for tRFP-non-normalized data, see Supplementary Figure 2B,C).

Together, these results suggested that the KA-induced *Pcdh11x* mRNA upregulation (Figures 1B,C) lead to an increased PCDH11X protein expression in the dentate gyrus, and this PCDH11X enrichment was GC-dependent. In addition, related to *Pcdh11x* KO but irrespective of PCDH11X labeling, an increased GCL dispersion in the *Pcdh11x*^KO+KA^ dentate gyrus become apparent (see Figure 3E and below).

### Impact of *Pcdh11x* KO on mossy fiber sprouting

**FIGURE 4:**
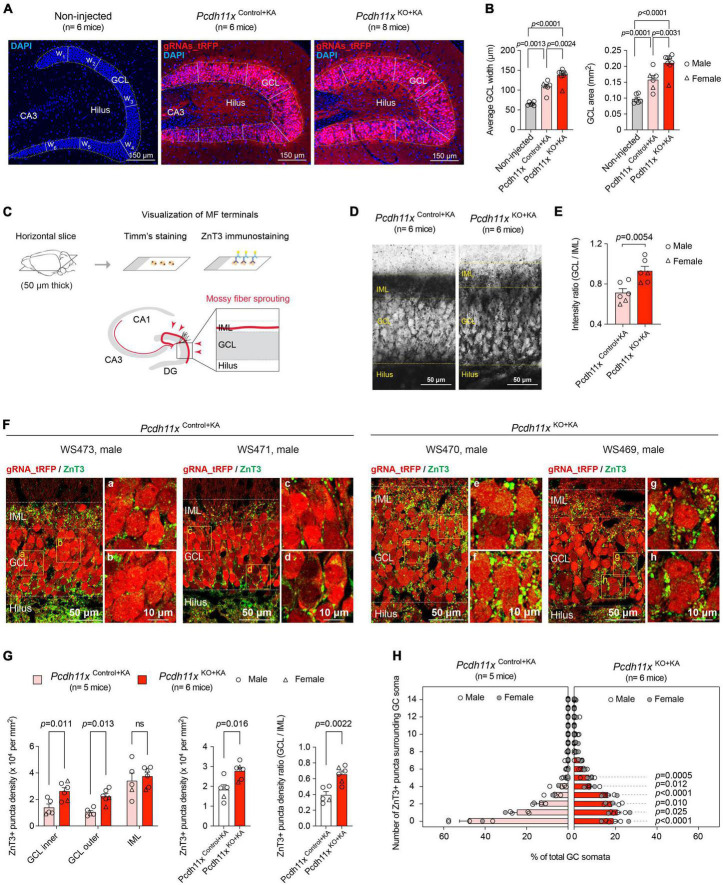
Impact of *Pcdh11x* KO on MF sprouting. **(A)** Confocal images show DAPI immunostaining and virally-delivered tRFP signal in the dentate gyrus of non-injected control, *Pcdh11x*^Control+KA^, and *Pcdh11x*^KO+KA^ mice. In each sample, the width of GCL was determined as the average of six width measurement (w_1_ to w_6_) based on DAPI staining. **(B)** Left plot shows quantification of average GCL width in non-injected, *Pcdh11x*^Control+KA^, and *Pcdh11x*^KO+KA^ samples. The transfected area was localized based on the tRFP signal. Right plot shows quantification of GCL area (quantified as the circumference of DAPI staining) in non-injected, *Pcdh11x*^Control+KA^, and *Pcdh11x*^KO+KA^ samples. Each data point represents one animal (one-way ANOVA tests, *F*(2,17) = 29, *p* < 0.0001; *p*-values of the *post hoc* analyses are indicated in the figure). **(C)** Experimental design for visualizing MF boutons by Timm’s staining and ZnT3 immunostaining. **(D)** Timm’s staining shows stratification of MF boutons in *Pcdh11x*^Control+KA^ and *Pcdh11x*^KO+KA^ mice. **(E)** Quantification of Timm’s signal intensity between GCL and IML in *Pcdh11x*^Control+KA^ and *Pcdh11x*^KO+KA^ mice (Welch’s *t*-test). **(F)** ZnT3 staining shows stratification of MF boutons in *Pcdh11x*^Control+KA^ and *Pcdh11x*^KO+KA^ mice. Areas highlighted with boxes are shown in higher magnification in panels **(a–h)**. **(G)** Quantification of ZnT3 + puncta density in the inner and outer half of GCL and in IML [left plot; two-way ANOVA, F_*Layer*_ (2,18) = 31, *p* < 0.0001; F_*KO*_ (1,9) = 7.8, *p* = 0.021; F_*Layer  x  KO*_ (2,18) = 1.9, *p* = 0.18; *post hoc* analyses: *Pcdh11x*^Control+KA^ vs *Pcdh11x*^KO+KA^, GCL inner: *p* = 0.01; GCL outer: *p* = 0.013; IML: ns, *p* = 0.46], in GCL and IML together (middle plot; Welch’s *t*-test, *p* = 0.016), and the GCL/IML ratio of ZnT3 + puncta density (right plot; Welch’s *t*-test, p = 0.0022). **(H)** Distribution of GC somata (in %) that are surrounded by 0, 1, 2, …, 14 ZnT3 + boutons in *Pcdh11x*^Control+KA^ and *Pcdh11x*^KO+KA^ mice (two-way ANOVA, F_#  of  puncta_ (14,126) = 141, *p* < 0.0001; F_*KO*_ (1,9) = 2.8, *p* = 0.13; F_#  of  puncta  x  KO_ (14,126) = 26, *p* < 0.0001; p-values of the *post hoc* analysis are indicated in the figure; *p*-values are > 0.05 for 6 or more puncta).

To study the KA-induced phenotypes in *Pcdh11x* KO, we employed the same experimental approach as described above (Figure 3D). First, we analyzed GCL dispersion, which is although mechanistically independent from MF sprouting (Haas et al., 2002; Heinrich et al., 2006; Duveau et al., 2011), a known phenotype of KA injections in the dentate gyrus. While KA-induced GCL dispersion developed both in *Pcdh11x*^Control+KA^ and *Pcdh11x*^KO+KA^, it was more pronounced in KOs (GCL width: non-injected, 66 ± 1.8 μm, *Pcdh11x*^Control+KA^, 109 ± 6.2 μm, *Pcdh11x*^KO+KA^, 138 ± 5.9 μm; one-way ANOVA, *Pcdh11x*^Control+KA^ vs *Pcdh11x*^KO+KA^, *p* = 0.0031; GCL area: non-injected, 0.097 ± 0.005 mm^2^, *Pcdh11x*^Control+KA^, 0.16 ± 0.013 mm^2^, *Pcdh11x*^KO+KA^, 0.21 ± 0.011 mm^2^, Figures 4A,B).

Next, we examined the impact of *Pcdh11x* KO on MF sprouting. To visualize MF sprouting, we utilized two MF labeling approaches: Timm’s staining and ZnT3 immunostaining (Figure 4C) (Luo et al., 2021). Using Timm’s staining, we observed dense signal in IML of *Pcdh11x*^Control+KA^, which is the typical targeting zone of MF sprouting. By contrast, the Timm’s signal became more diffuse overall but also denser in GCL of *Pcdh11x*^KO+KA^, highlighting a pattern atypical for MF sprouting. To quantify these observations, we measured the signal intensity ratio between GCL and IML, which was significantly higher in KOs than in controls (GCL/IML signal ratio: *Pcdh11x*^KO+KA^, 0.93 ± 0.046, n = 6 mice; *Pcdh11x*^Control+KA^, 0.71 ± 0.04, n = 6 mice; Welch’s *t*-test, *p* = 0.0054) (Figures 4D,E). ZnT3 immunostaining confirmed this pattern. A large number of ZnT3 + puncta were present in the IML of both *Pcdh11x*^Control+KA^ and *Pcdh11x*^KO+KA^, but become significantly enriched in the GCL of *Pcdh11x*^KO+KA^ compared to *Pcdh11x*^Control+KA^ (Figure 4F), suggesting that MF sprouting target specification was altered in KOs.

To further study this phenotype, we first considered the possibility that the apparent change in target specificity appeared as a consequence of increased GCL dispersion in KOs. According to this scenario, sprouting MF axons in KOs populated the same spatial area as in controls, but the broader GC dispersion created an altered context. To test this possibility, we quantified ZnT3 + puncta density in the inner (proximal to hilus) and outer (proximal to IML) half of GCL, and in IML. We hypothesized that ZnT3 + puncta density would not change in the inner half of GCL if the effect was due to increased GCL dispersion, because the inner half of GCL in KOs remained before the GCL/IML border seen in controls. However, ZnT3 + puncta density was significantly increased both in the inner and outer half of GCL in KOs compared to controls, whereas that in IML was similar in both conditions (*Pcdh11x*^Control+KA^: GCL inner: 1.4 ± 0.26 × 10^4^ puncta/mm^2^, GCL outer: 1.0 ± 0.13 × 10^4^ puncta/mm^2^, IML: 3.4 ± 0.58 × 10^4^ puncta/mm^2^, n = 5 mice; *Pcdh11x*^KO+KA^: GCL inner: 2.6 ± 0.27 × 10^4^ puncta/mm^2^, GCL outer: 2.2 ± 0.21 × 10^4^ puncta/mm^2^, IML: 3.8 ± 0.31 × 10^4^ puncta/mm^2^, n = 6 mice) (Figure 4G), suggesting that target specificity in KOs has changed independently of GCL dispersion. Consequently, the total (as measured in the inner and outer half of GCL, and IML) ZnT3 + puncta density (*Pcdh11x*^Control+KA^: 1.8 ± 0.25 × 10^4^ puncta/mm^2^, n = 5 mice; *Pcdh11x*^KO+KA^: 2.8 ± 0.17 × 10^4^ puncta/mm^2^, n = 6 mice) and the GCL/IML ZnT3 + puncta density ratio increased in KOs (*Pcdh11x*^Control+KA^: 0.39 ± 0.044, n = 5 mice; *Pcdh11x*^KO+KA^: 0.66 ± 0.043, n = 6 mice) (Figure 4G).

To gain further insights into the target specification of MF sprouting, we quantified the number of ZnT3 + puncta surrounding GC somata as a proxy for potential synapses. In *Pcdh11x*^Control+KA^, we found that ∼50% of GCs somata were lacking adjacent ZnT3 + puncta. By contrast, in *Pcdh11x*^KO+KA^, only ∼20% of GCs somata were lacking adjacent ZnT3 + puncta while the rest were surrounded with more ZnT3 + puncta than those in controls (Figure 4H).

### Electrophysiological characterization of *Pcdh11x* KO GCs

**FIGURE 5:**
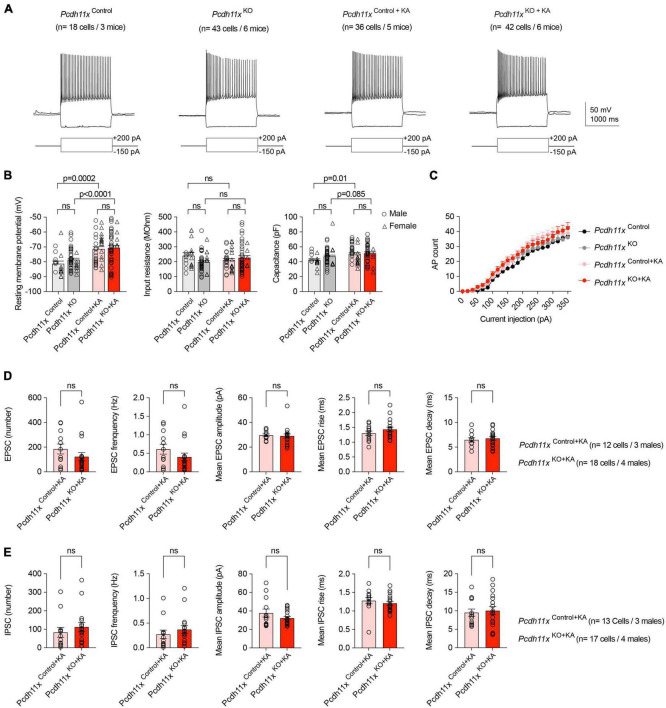
Electrophysiological characterization of *Pcdh11x*^Control+KA^ and *Pcdh11x*^KO+KA^ GCs. **(A)** Example electrophysiological traces show responses to 1.5 s long current pulse injections in *Pcdh11x*^Control^, *Pcdh11x*^KO^, *Pcdh11x*^Control+KA^, and *Pcdh11x*^KO+KA^ GCs. **(B)** Quantification of resting membrane potential, input resistance, and capacitance (two-way ANOVA tests; resting membrane potential: F_*KA  treatment*_ (1,135) = 30, *p* < 0.0001; F_*KO*_ (1, 135) = 1.1, *p* = 0.30; F_*KA  treatment  x  KO*_ (1,135) = 0.35, *p* = 0.56; input resistance: F_*KA  treatment*_ (1,135) = 0.0009, *p* = 0.98; F_*KO*_ (1, 135) = 0.92, *p* = 0.33; F_*KA  treatment  x  KO*_ (1,135) = 4.3, *p* = 0.04; capacitance: F_*KA  treatment*_ (1,135) = 9.8, *p* = 0.0022; F_*KO*_ (1, 135) = 1.8, *p* = 0.18; F_*KA  treatment  x  KO*_ (1,135) = 1.1, *p* = 0.30; *p*-values of *post hoc* analyses are indicated in the figure; data points represent single cells). **(C)** Quantification of steady-state current injection-evoked action potential (AP) counts. **(D)** Quantification of EPSC parameters recorded from *Pcdh11x*^Control+KA^ and *Pcdh11x*^KO+KA^ GCs (Mann–Whitney U test; data points represent single cells recorded from males). **(E)** Quantification of IPSC parameters recorded from *Pcdh11x*^Control+KA^ and *Pcdh11x*^KO+KA^ GCs (Mann-Whitney *U* test; data points represent single cells recorded from males).

Next, following the same injection schedule as above, we made patch-clamp recordings from *Pcdh11x*^Control+KA^ and *Pcdh11x*^KO+KA^ GCs. As additional controls, we also included GCs from *Pcdh11x*^Control^ and *Pcdh11x*^KO^ (six weeks after gRNA injection), neither of which received KA (Figure 5A). The resting membrane potential (RMP), input resistance (R), and capacitance (C) of cells reflected consequences of KA, but not gRNA treatment (for *Pcdh11x*^Control^, n = 18 cells/*Pcdh11x*^KO^, n = 43 cells/*Pcdh11x*^Control+KA^, n = 36 cells/*Pcdh11x*^KO+KA^, n = 42 cells, respectively; RMP (mV): −81 ± 1.80/−79 ± 1.0/−71 ± 1.6/−71 ± 1.7; R (MOhm): 240 ± 20/198 ± 10/211 ± 11/226 ± 14; C (pF): 42 ± 1.8/47 ± 1.8/51 ± 2.0/52 ± 1.8) (Figure 5B). In addition, we analyzed action potential firing threshold, amplitude, and attenuation, none of which showed difference between the different conditions (not shown). Further, steady-state current injection-evoked action potential (AP) counts did not differ between the groups (Figure 5C). These results established that *Pcdh11x* KO had no effect on the intrinsic electrophysiological properties of GCs. Next, we analyzed spontaneous glutamatergic EPSCs (in presence of 10 μM Gabazine) and GABAergic IPSCs (in presence of 10 μM APV and 5 μM NBQX) in *Pcdh11x*^Control+KA^ and *Pcdh11x*^KO+KA^ GCs. We hypothesized that somatic boutons in *Pcdh11x* KOs may elicit larger and/or faster synaptic events, because they were closer to the recording pipette. However, neither the number and frequency of recorded EPSCs and IPSCs, nor their amplitudes, rise and decay times revealed significant differences between the two groups (Figures 5D,E). This could be because synaptic events evoked by sprouted synapses were not sufficiently represented in our recordings (e.g., they were not spontaneously activated or possibly represented silent synapses), or the recordings did not have sufficient resolution for differences, or both.

### Morphological characterization of *Pcdh11x* KO GCs

**FIGURE 6:**
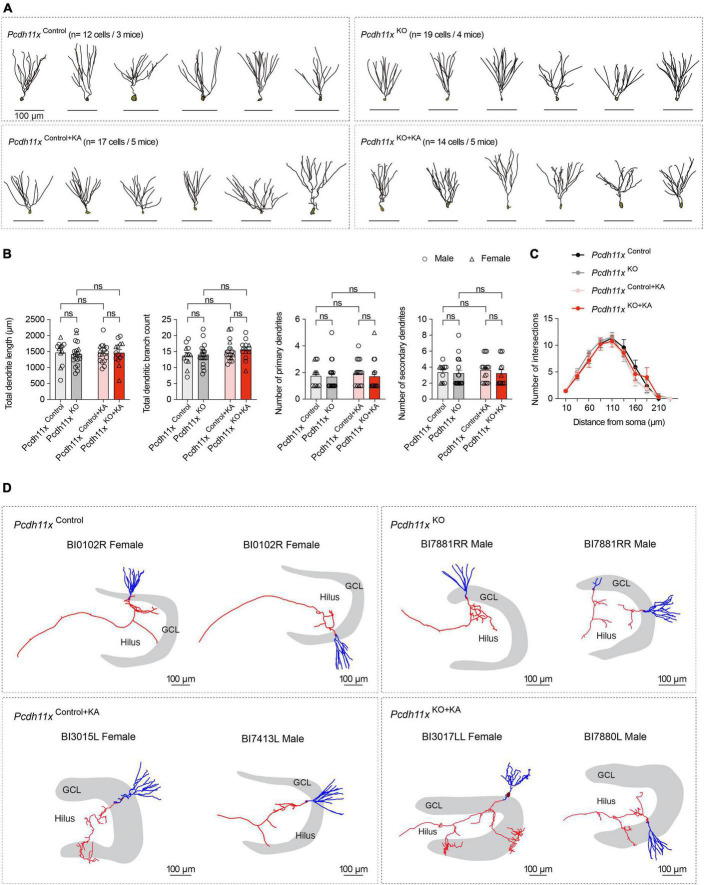
Morphological characterization of *Pcdh11x*^Control+KA^ and *Pcdh11x*^KO+KA^ GCs. **(A)** Morphological reconstruction of dendrites from *Pcdh11x*^Control^, *Pcdh11x*^KO^, *Pcdh11x*^Control+KA^, and *Pcdh11x*^KO+KA^ GCs. **(B)** Quantification of dendritic parameters, such as total dendrite length, total dendritic branch count, number of primary dendrites, and number of secondary dendrites (two-way ANOVA test; data points represent single cells). **(C)** Sholl analysis of dendritic complexity. None of the comparisons had significance *p* < 0.05 (two-way ANOVA test). **(D)** Morphological reconstruction of axons from *Pcdh11x*^Control^, *Pcdh11x*^KO^, *Pcdh11x*^Control+KA^, and *Pcdh11x*^KO+KA^ GCs. Axons and dendrites are shown in red and blue respectively. For further examples, see Supplementary Figure 3.

To study the morphology of individual GCs, we filled cells with biocytin during the patch-clamp recordings and reconstructed them afterwards. A limitation of this approach, however, is that the recovery of axons (e.g. in CA3 or sprouted fibers in IML) is limited in brain slice preparation. First, we analyzed dendritic morphology (Figure 6A), because the overexpression and knockdown of *Pcdh11x* was previously reported to reduce and increase dendritic complexity in developing neurons, respectively (Wu et al., 2015). However, neither the total dendritic length, total dendritic branch count, number of primary and secondary dendrites, nor Sholl analysis showed a difference between *Pcdh11x*^Control^, *Pcdh11x*^KO^, *Pcdh11x*^Control+KA^, and *Pcdh11x*^KO+KA^ GCs (Figures 6B,C). Second, whenever possible, we reconstructed axons from GCs. As expected, GCs in the KA-non-injected control groups (*Pcdh11x*^Control^ and *Pcdh11x*^KO^ GCs) lacked axons in GCL or IML. By comparison, GCs in both KA-injected groups (*Pcdh11x*^Control+KA^ and *Pcdh11x*^KO+KA^ GCs) displayed MF sprouting, i.e., axons were detectable in GCL and to some extent in IML (Figure 6D and Supplementary Figure 3). While insights into target specification by this analysis were limited, it confirmed the presence of *Pcdh11x*^KO+KA^ GC axons in GCL.

### Immuno-electron microscopy characterization of sprouted *Pcdh11x* KO GC synapses

**FIGURE 7:**
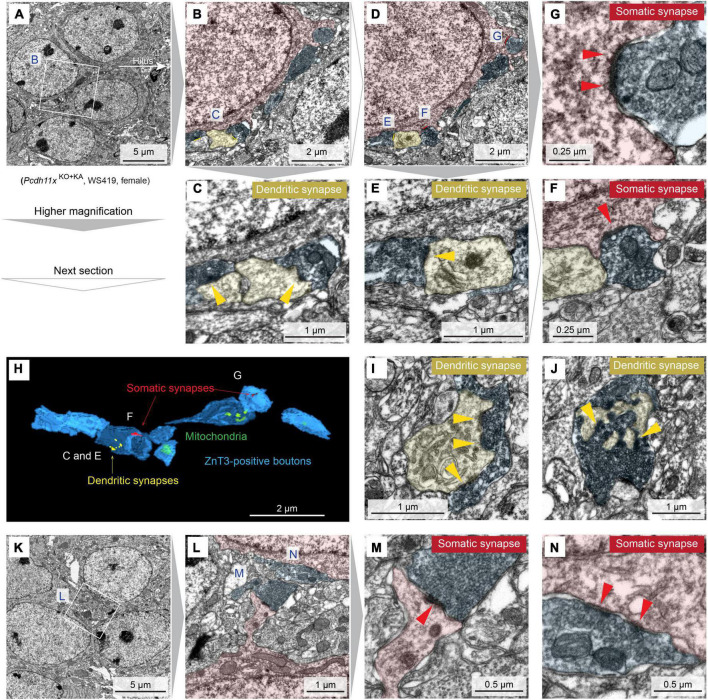
Immuno-electron microscopy characterization of *Pcdh11x*^KO+KA^ GC synapses. **(A)** Transmission electron microscopy image of four GC somata in GCL. In white box, ZnT3 + boutons are visible next to GC soma. **(B)** Magnification of the white box in panel A. GC soma, dendrite, and presynaptic ZnT3 + boutons are presudo-colored in red, yellow, and blue, respectively. **(C)** Magnification of the area labeled with C in panel B shows synapses on dendrites (yellow arrowheads). **(D)** Image shows the next section of what is shown in panel **(B)**. **(E–G)** Magnification of the areas labeled with E, G, and F in panel **(D)** show somatic, somatic and dendritic, and dendritic synapses, respectively (yellow and red arrowheads). In the lower right part of panel **(F)**, a dendritic synapse is also visible. However, the presynaptic compartment is lacking ZnT3 and thus MF identity of this synapse could not be confirmed. **(H)** 3D reconstruction of ZnT3 + boutons and synapses shown in panels **(A–F)**. **(I,J)** Images show additional dendritic synapses with multiple release sites (yellow arrowheads) from the same animal. **(K)** Image show five GC somata in GCL. **(L)** Magnification of the area shown in panel **(K)**. **(M,N)** Magnification of the areas labeled with M and N in panel **(L)** show somatic synapses with one and multiple release sites, respectively (red arrowheads).

Thus far, our histological analyses revealed differences in target specificity between *Pcdh11x* controls and KOs after MF sprouting, but our electrophysiological and morphological analyses could not further substantiate this. Importantly, the question whether ZnT3 + and Timm + boutons in GCL formed synapses remained open. To answer this question, we prepared horizontal sections from the ventral dentate gyrus, immunostained them with ZnT3 antibody and used immuno-electron microscopy (Figure 7 and Supplementary Figure 4). In *Pcdh11x*^Control+KA^, we only found dendritic synapses, an expected outcome after KA treatment (Supplementary Figure 4). By contrast, in *Pcdh11x*^KO+KA^, electron microscopy revealed an abundance of ZnT3 + synapses on GC somata (7G,F,M,N and Supplementary Figure 4). The synapses contained one or multiple release sites and many vesicles. In some cases, ZnT3 + boutons formed synapses both on soma and neighboring dendrites in GCL (Figure 7H) or only on dendrites (Figures 7I,J and Supplementary Figure 4). Such dendrites in GCL may have belonged to GCs whose soma was proximal to hilus or interneurons (Frotscher et al., 2006).

## Discussion

Hippocampal MF sprouting is a striking example of circuit formation in the adult brain (Seng et al., 2022). Previously, we studied the induction mechanisms of MF sprouting (Luo et al., 2021). Here, we investigated the question of target specificity.

Associated with MF sprouting, CAM expression and/or abundance changes have been previously described in different models, such as pilocarpine (PC) or kainate (KA) induced status epilepticus and intrahippocampal electrical stimulation (IES). Arguably, the most striking phenotype was achieved by the knockdown of *Unc5a*, which prevented PC-induced recurrent MF sprouting in hippocampal slice cultures (Muramatsu et al., 2010), directly implicating this molecule in axon guidance. Others reported up-regulation of *Nrcam*, *Slit1*, *Celsr3*, *Sema6a*, *Epha4*, and *Epha7* transcripts in whole hippocampal tissue (PC model) (Hansen et al., 2014), increased protein abundance of N-cadherin (Cdh2) (PC model) (Shan et al., 2002) and NCAM (encoded by *Ncam1* or *Ncam2*) (KA model) (Niquet et al., 1993) in sprouted MF synapses, and transient down-regulation of *Sema3a* (IES model) (Holtmaat et al., 2003). In addition, C1q-like-s were characterized in MF sprouting. Typically, C1QL1 and C1QL3 are secreted from MF synapses and form a complex with presynaptic NRXN3 to facilitate trans-synaptic recruitment of kainate-sensitive glutamate receptors (KARs) in CA3 cells (Matsuda et al., 2016). While MF sprouting still developed in the double *C1ql2*/*C1ql3* knock-out mice, KARs were not recruited to sprouted synapses (PC model; Matsuda et al., 2016), showing a shared feature between naive and sprouted MF synapses.

Together, these findings illuminated a complex landscape behind MF sprouting. As a caveat, most observations were made in tissue-level samples and/or after seizures, limiting delineation of cell types in which CAM abundance has changed and causal dependencies (e.g., if CAM changes were required for sprouting or induced by seizures). We alleviated these limitations by specifically studying non-sprouting and sprouting GCs (1 and 14 days after KA), sampled before the expected onset of status epilepticus (typically 14–28 days after KA) (Tanaka et al., 1992; Ben-Ari and Cossart, 2000). Our results did not reveal significant transcriptomic changes in the above listed molecules, with the one exception of *Slit1*. However, in contrast to up-regulation at the tissue level (Hansen et al., 2014), we found down-regulation of *Slit1* in KA GCs (Figure 1). It is possible that (i) previously reported molecules did not change in GCs (but in other cell types), (ii) their expression changed in GCs but at other time points as in our study, (iii) they were induced by status epilepticus, (iv) they manifested themselves only at protein level (e.g., N-cadherin, NCAM), or (v) they were model specific. Thus, we focused on differentially expressed CAMs that were upregulated in our single GC data set, and shortlisted *Fat3*, *Pcdh11x*, and *Cntn4* for a CRISPR/Cas9-based *in vivo* screen. Sequence analysis revealed loss-of-function deletions in *Pcdh11x*, but not in *Fat3* or *Cntn4*, which were not considered further in this study.

With regard to *Pcdh11x*, we showed upregulation of *Pcdh11x* mRNA and enrichment of PCDH11X protein in *Pcdh11x* non-deficient control GCs during MF sprouting. Furthermore, using a CRISPR/Cas9-based strategy *in vivo*, we showed that while MF sprouting still developed in *Pcdh11x* KOs (*Pcdh11x*^KO+KA^), (i) GC dispersion was increased, (ii) sprouted synapses frequently formed on GC somata in addition to dendrites, and (iii) ∼50% more ZnT3 + puncta were detectable in GCL compared to *Pcdh11x* non-deficient controls (Figures 1–4, 7 and Supplementary Figure 4).

*Pcdh11x* was previously implicated in dendritic branching in developing neurons (Wu et al., 2015) and in the differentiation and proliferation of neural stem cells (Zhang et al., 2014). However, it is unlikely that these functions contributed to the phenotypes. First, dendritic branching was not different between *Pcdh11x*^Control+KA^ and *Pcdh11x*^KO+KA^ GCs (Figure 6), and second, although the adult dentate gyrus retains a neurogenic niche producing GCs, the use of *Calb1*^Cre/+^ line and adeno associated virus (AAV) ensured that *Pcdh11x* mutations occurred only in mature GCs but not in neural stem cells (Brandt et al., 2003).

**FIGURE 8:**
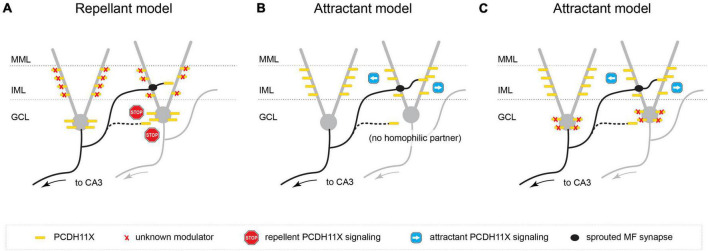
Possible models for PCDH11X function in MF sprouting. **(A)** PCDH11X is surface-expressed on sprouting GC axons as well as in both the IML and GCL, and homophilic *trans* PCDH11X interactions serve as a repellant in both layers. This model requires a second unknown modulator that selectively negates repellant PCDH11x signaling in IML. **(B)** PCDH11X is an attractant and is surface displayed on sprouting GC axons as well as GC dendrites in IML. Homophilic PCDH11X interactions drive selective synapse targeting to the IML during sprouting. **(C)** PCDH11X is an attractant and is surface displayed on sprouting GCL axons as well as on somata and dendrites both in GCL and IML. This model requires a second unknown modulator that competes for binding with PCDH11X and outcompetes homophilic *trans* interactions.

Alternatively, *Pcdh11x* also has been implicated in homophilic *trans* cell-cell interactions (Harrison et al., 2020; Pancho et al., 2020). In a first potential hypothesis, PCDH11X is surface-expressed on sprouting GC axons (ZnT3 + MF boutons) as well as in both the IML and GCL, and homophilic *trans* PCDH11X interactions serve as a repellant or inhibit synapse targeting in both layers. This model is consistent with the phenotype we observed in the *Pcdh11x* KOs, where KA-induced sprouting occurs not only in the IML (as seen in controls) but in the GCL as well—presumably, according to this model, due to a release of PCDH11X-mediated inhibition of synapse targeting in the GCL. However, in order to achieve the IML-specific targeting seen in KA-treated wildtypes, this model requires that either (a) PCDH11X is exclusively expressed in GCL, which our immunostaining data shows not to be the case, or (b) a second unknown modulator selectively negates repellant PCDH11x signaling or releases inhibition of synapse targeting in the IML (Figure 8A).

A second, simpler model assumes that PCDH11X is a strong attractive signal and is surface displayed on sprouting GC axons as well as GC proximal dendrites in the IML; by virtue of specific expression in the IML (as opposed to the GCL), homophilic PCDH11X interactions drive selective synapse targeting to the IML during sprouting. In apparent contradiction to the model, we observed PCDH11X immunostaining in both the IML and GCL, including somatic and proximal dendritic compartments of GCs; however, the antibody we used targeted the intracellular domain of PCDH11X and could conceivably label intracellularly-retained molecules instead of surface-displayed PCDH11X in the soma, therefore preserving the possibility of this second hypothesis. Assuming this model, we explain the targeting of sprouted MF synapses onto both GC somata and dendrites of *Pcdh11x*^KO+KA^ as follows: in the absence of a strong attractive signal that condenses or concentrates synapse targeting to the IML, synapses will form indiscriminately, in both the GCL and IML (Figure 8B).

Finally, a third model posits that PCDH11X—similarly to the second model—is an attractive cue. However, unlike the second model, it assumes that PCDH11X is expressed in both the GCL and/or GC cell bodies as well as in the IML and/or GC proximal dendrites, since we cannot rule out the possibility that the antibody used in our immunostaining experiments does indeed label surface-displayed PCDH11X molecules at the soma. Instead, it invokes a modulatory or repellant signal specific to the GCL, such as an unidentified CAM that competes for binding with PCDH11X (e.g., via repellant, heterophilic interactions that outcompete homophilic *trans* interactions) (Figure 8C).

While not in our focus, the lack of an attractant, homophilic *trans* PCDH11X interaction may also explain the increased GCL dispersion seen in *Pcdh11x* KOs. Although both GC dispersion and MF sprouting were induced by KA, they are mechanistically independent. GC dispersion is due to impaired Reelin secretion by Cajal-Retzius cells (Haas et al., 2002; Heinrich et al., 2006; Duveau et al., 2011), whereas MF sprouting is a GC autonomous process (Luo et al., 2021). It is plausible that *trans* binding of PCDH11Xs displayed on neighboring neurons/dendrites after KA could serve as a structural break before further dispersion in controls, but not in KOs.

Together, our results revealed that PCDH11X controls synapse targeting during MF sprouting. With regard to implications for epilepsy, partial *Pcdh11x* duplication (as part of a broader Xq13-q21 duplication) was reported in one patient with recurrent seizures (Linhares et al., 2016). Based on the association of other protocadherins with epilepsy, such as *Pcdh19* (Dibbens et al., 2008; also see Hoshina et al., 2021) and *Pcdh7* (Lal et al., 2015), authors of the partial *Pcdh11x* duplication study hypothesized that the *Pcdh11x* mutation may be relevant for the seizures of this patient (Linhares et al., 2016). Our results provide additional insights showing altered connectivity in *Pcdh11x* KO. However, a more thorough testing of a link between *Pcdh11x* mutations and seizures, including conditions under which somatic synapses become activated in the brain, is beyond the scope of our study. Further, it is also clear that other CAMs may be involved in MF sprouting which our study did not cover. As such, the roles of SLIT1, FAT3, and CNTN4, if any, remain to be determined. More broadly, delineating which signals control target cell type selectivity (among GCs and different GABAergic interneuron types) as postsynaptic targets remain a major challenge. MF sprouting is a robust model to study these questions. Potentially, at least some of the mechanisms will be generalizable beyond MF sprouting, and applicable in other cell types in which to facilitate circuit repair in brain disorders and after injuries.

## Data availability statement

The original contributions presented in this study are included in the article/Supplementary material, further inquiries can be directed to the corresponding author.

## Ethics statement

The animal study was reviewed and approved by Veterinary Office of Zurich Kanton.

## Author contributions

WL and CF designed research and wrote the manuscript. WL, NC-O, CS, ME, DL, and TL performed research and analyzed data. All authors contributed to the article and approved the submitted version.
