# Supplementary material for: Pcdh11x controls target specification of mossy fiber sprouting
Source: Front Neurosci. 2022 Sep 1;16:888362. doi: 10.3389/fnins.2022.888362 (PMC9475199; doi:10.3389/fnins.2022.888362)
Supplement: Supplementary file 1 [file Data_Sheet_1.pdf]

Supplementary Material for

Pcdh11x controls target specification of mossy fiber sprouting

This file includes:

Supplementary Figures (Figure S1-S4)

List of Supplementary Figures:

Figure S1	Genomic targeting of CAMs.
Figure S2	PCDH11x protein levels in the dentate gyrus.
Figure S3	Morphological characterization of *Pcdh11x* ^{KO} and *Pcdh11x* $^{KO+KA}$ GCs.
Figure S4	Immuno-electron microscopy characterization of *Pcdh11x* $^{Control+KA}$ and *Pcdh11x* $^{KO+KA}$ GC synapses.

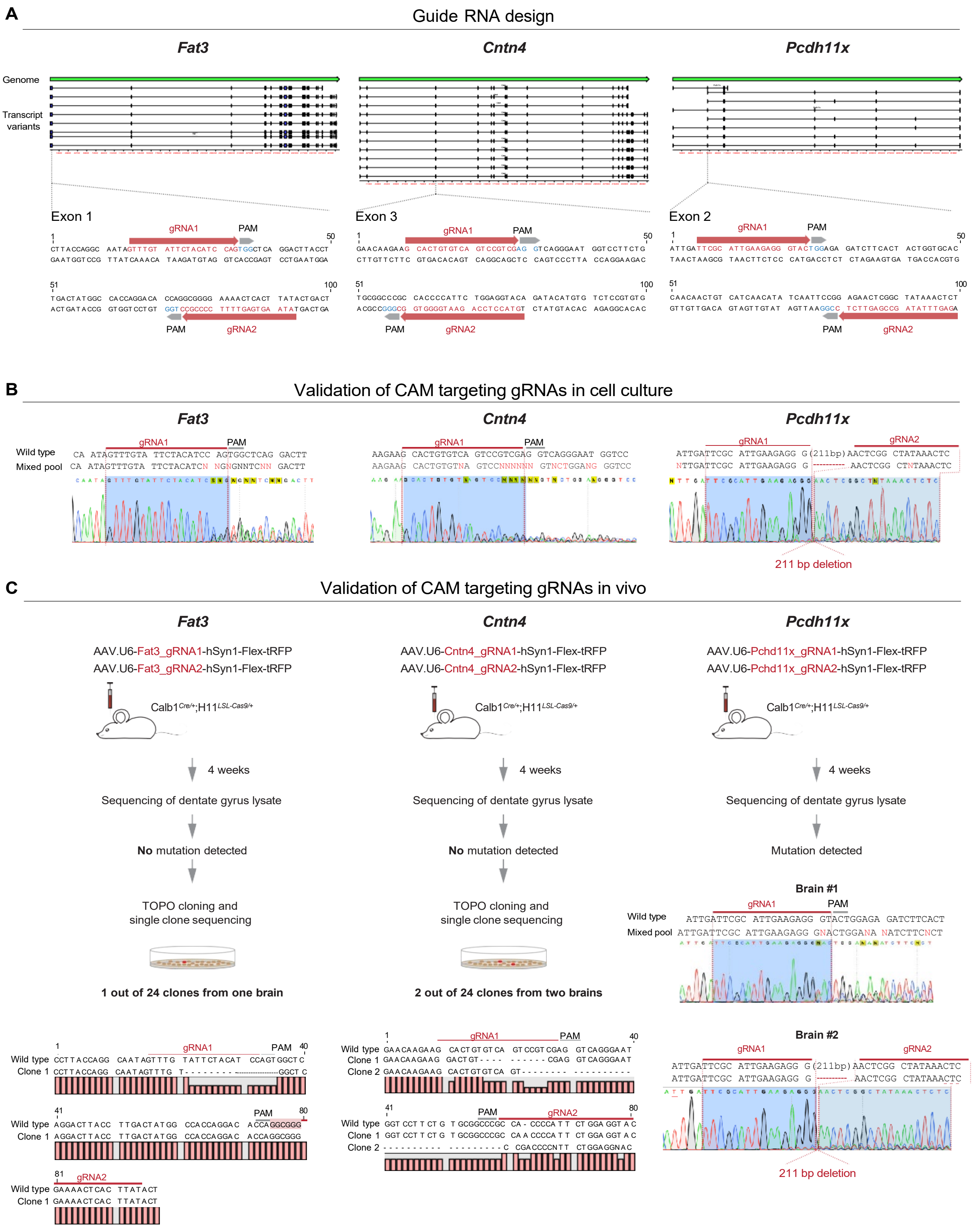
A
Guide RNA design
Fat3
Cntn4
Pcdh11x
Genome
Transcript variants
Exon 1
Exon 3
Exon 2
gRNA1
PAM
gRNA2
B
Validation of CAM targeting gRNAs in cell culture
Wild type
Mixed pool
211 bp deletion
C
Validation of CAM targeting gRNAs in vivo
AAV.U6-Fat3_gRNA1-hSyn1-Flex-tRFP
AAV.U6-Fat3_gRNA2-hSyn1-Flex-tRFP
AAV.U6-Cntn4_gRNA1-hSyn1-Flex-tRFP
AAV.U6-Cntn4_gRNA2-hSyn1-Flex-tRFP
AAV.U6-Pcdh11x_gRNA1-hSyn1-Flex-tRFP
AAV.U6-Pcdh11x_gRNA2-hSyn1-Flex-tRFP
Calb1Cre/+;H11LSL-Cas9/+
4 weeks
Sequencing of dentate gyrus lysate
No mutation detected
Mutation detected
TOPO cloning and single clone sequencing
1 out of 24 clones from one brain
2 out of 24 clones from two brains
Brain #1
Brain #2
Clone 1
Clone 2

Figure S1. Genomic targeting of CAMs. (A) For each of the shortlisted genes, two gRNAs were designed to target early exons shared among different transcript isoforms. **(B)** The efficacy of gRNA targeting was validated in Cas9-expressing Neuro-2a mouse cell lines by mixed pool sequencing for each gene separately. Mutations and/or deletions are shown in red in „Mixed pool“ together with Sanger chromatograms. **(C)** Experimental design and schedules used for validation of gene targeting in Cas9-expressing mice *in vivo*. For *Fat3* and *Cntn4*, mutations and/or deletions could not be detected from dentate gyrus lysates; mutations and/or deletions are shown at the bottom for the only 1 and 2 clones, respectively, in which they could be detected after TOPO cloning. For *Pcdh11x*, mutations and/or deletions could be detected from dentate gyrus lysates. Sanger chromatograms are show at the bottom.

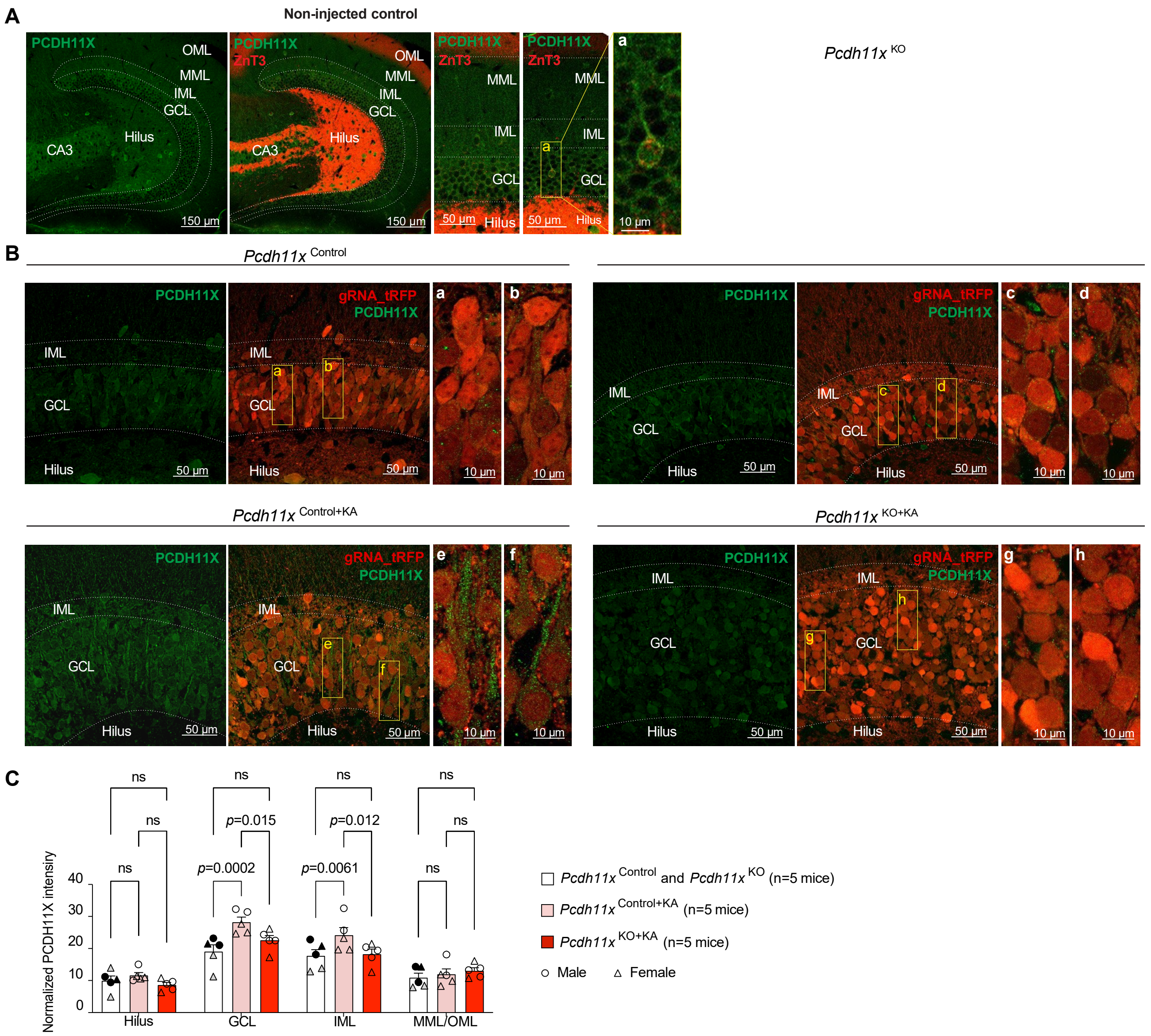

Figure S2. PCDH11x protein levels in the dentate gyrus. (A) Confocal images show PCDH11X and ZnT3 immunostaining in non-injected naive animals. The third and fourth panels (from left) show higher magnification images from GCL, IML and MML. Panel (a) shows a higher magnification image of a PCDH11X positive control GC. **(B)** PCDH11X expression in the dentate gyri of *Pcdh11x* ^{Control}, *Pcdh11x* ^{KO}, *Pcdh11x* $^{Control+KA}$ and *Pcdh11x* $^{KO+KA}$ mice 14 days after KA injection. Areas highlighted with yellow rectangles (a-h) are shown at higher magnification on the right. tRFP (gRNA_tRFP) was virally delivered to the cells. **(C)** Quantification of PCDH11X protein level in *Pcdh11x* ^{Control}, *Pcdh11x* ^{KO}, *Pcdh11x* $^{Control+KA}$ and *Pcdh11x* $^{KO+KA}$ dentate gyri. Unlike in **Fig. 3F**, these values were not normalized to the tRFP signal seen in the GFP channel (see also main text and **Materials and Methods**). As KA non-injected controls, data from two *Pcdh11x* ^{Control} (grey triangle) and three *Pcdh11x* ^{KO} (black circle and black triangle) were pooled together (two-way ANOVA; F_{Layer} (3,36)=106, p<0.0001; $F_{treatment}$ (2,12)=3.3, p=0.07; $F_{Layer\ x\ Treatment}$ (6,36)=4.4, p=0.0018; p-values of post-hoc analyses are shown in the plot).

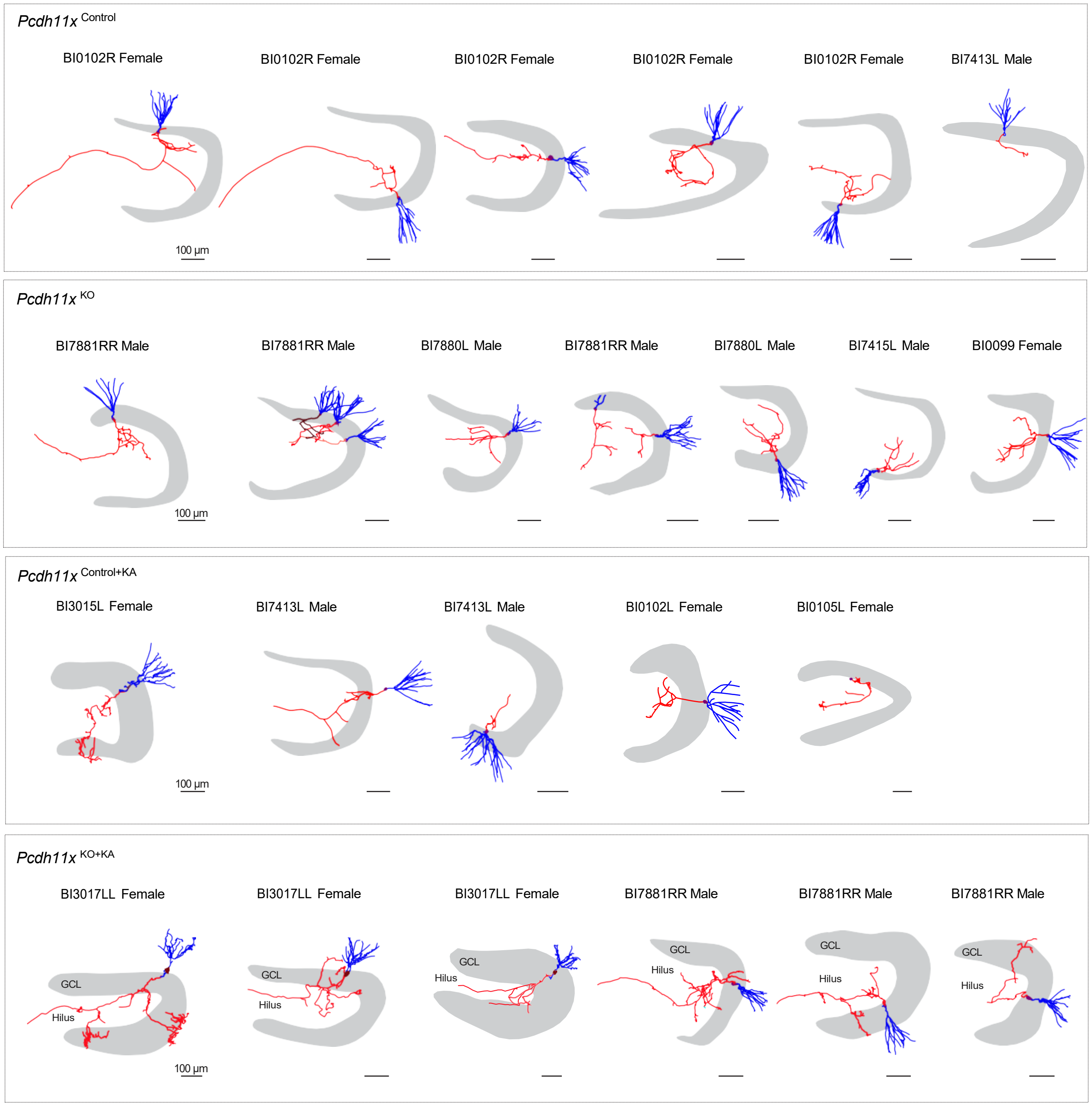

Figure S3. Morphological characterization of *Pcdh11x* ^{KO} and *Pcdh11x* $^{KO+KA}$ GCs. Morphological reconstruction of *Pcdh11x* ^{Control}, *Pcdh11x* ^{KO}, *Pcdh11x* $^{Control+KA}$, and *Pcdh11x* $^{KO+KA}$ GCs. Axons and dendrites are shown in red and blue respectively. GCL is highlighted with gray. The animal number and male/female information are indicated above each plot. For BI7881RR (*Pcdh11x* ^{KO}), the axons of three different GCs are colored with dark red, red, and light red for clarity.

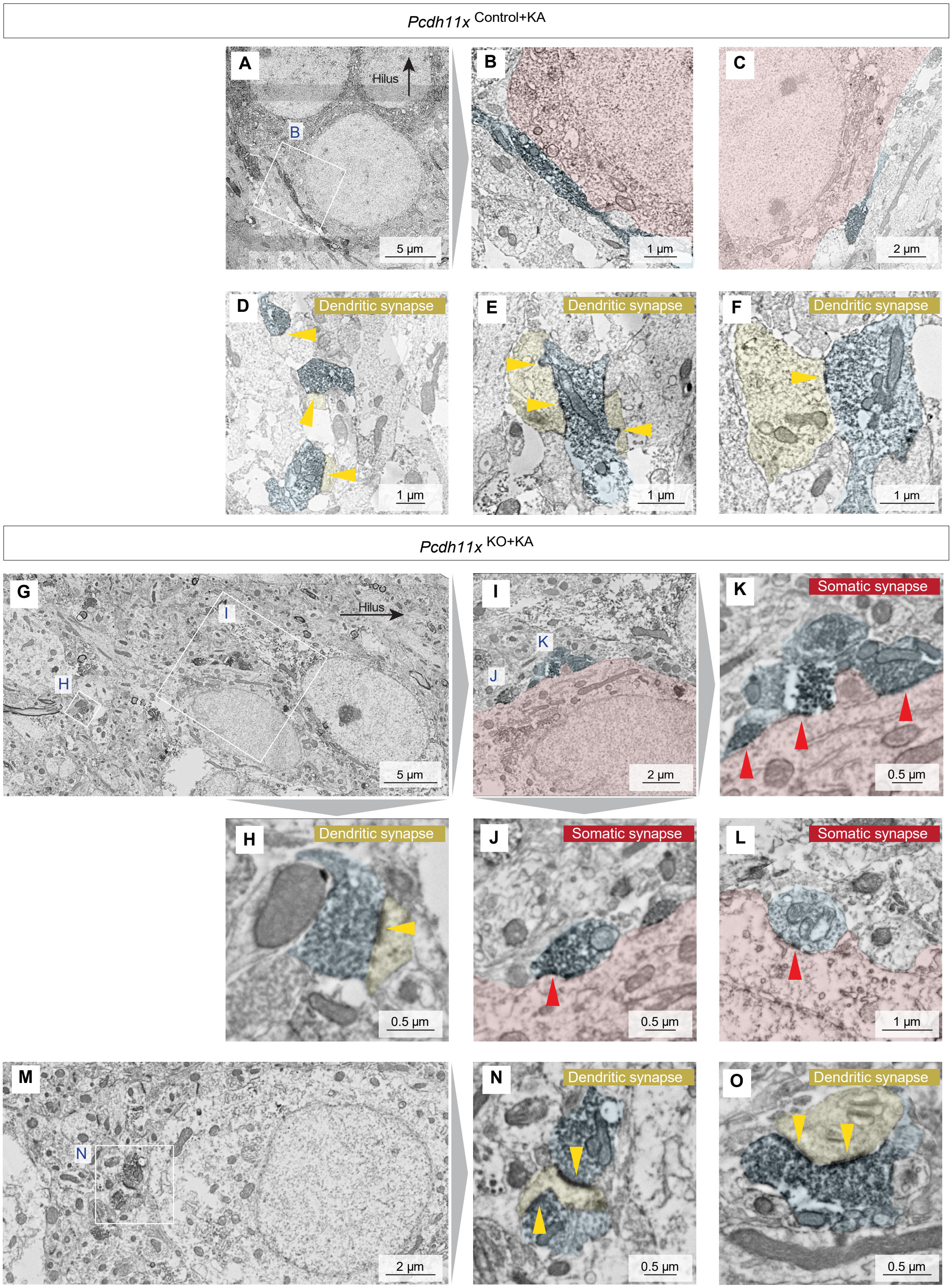

Pcdh11x Control+KA
A
Hilus
B
5 µm
B
1 µm
C
2 µm
D
Dendritic synapse
1 µm
E
Dendritic synapse
1 µm
F
Dendritic synapse
1 µm
Pcdh11x KO+KA
G
Hilus
I
H
5 µm
I
K
J
2 µm
K
Somatic synapse
0.5 µm
H
Dendritic synapse
0.5 µm
J
Somatic synapse
0.5 µm
L
Somatic synapse
1 µm
M
N
2 µm
N
Dendritic synapse
0.5 µm
O
Dendritic synapse
0.5 µm

Figure S4. Immuno-electron microscopy characterization of *Pcdh11x* $^{Control+KA}$ and *Pcdh11x* $^{KO+KA}$ GC synapses. (A) Scanning electron microscopy image of three GC somata in GCL from a *Pcdh11x* $^{Control+KA}$ animal. In white box, a ZnT3+ process is visible next to GC soma. **(B)** Magnification of the area labeled with B in panel A. The GC soma and ZnT3+ processes are presudo-colored in red and blue, respectively. Synapses between the soma and ZnT3+ process could not be recognized. **(C)** Another example of a ZnT3+ process passing by a GC soma without forming synaptic contacts. **(D-F)** Images show ZnT3+ processes forming dendritic synapses in GCL of a *Pcdh11x* $^{Control+KA}$ animal. The ZnT3+ boutons and dendrites are presudo-colored in blue and yellow, respectively. **(G)** Image shows three GC somata in GCL from a male *Pcdh11x* $^{KO+KA}$ animal. **(H)** Magnification of the area labeled with H in panel G shows a ZnT3+ process forming a dendritic synapse. **(I)** Magnification of the area labeled with I in panel G shows ZnT3+ process forming synapses on GC soma. **(J-K)** Magnification of the areas labeled with J and K in panel I. **(L)** Another example of a somatic synapse in the same animal. **(M)** Electron microscopy image of one GC somata in GCL from a *Pcdh11x* $^{KO+KA}$ animal. **(N)** Magnification of the area labeled with N in panel M shows ZnT3+ processes forming dendritic synapses. **(O)** Another example of dendritic synapses from the same animal.
